# Construction of Carbon Nanofiber-Wrapped SnO_2_ Hollow Nanospheres as Flexible Integrated Anode for Half/Full Li-Ion Batteries

**DOI:** 10.3390/nano13152226

**Published:** 2023-07-31

**Authors:** Qi Shao, Jiaqi Liu, Xiantao Yang, Rongqiang Guan, Jing Yu, Yan Li

**Affiliations:** 1 School of Electrical and Information, Jilin Engineering Normal University, Changchun 130052, China; shaoqi727@163.com (Q.S.);; 2 School of Materials Science and Engineering, Changchun University of Science and Technology, Changchun 130022, China; liujiaqi_forsci@163.com; 3 School of Metallurgy, Northeastern University, Shenyang 110819, China

**Keywords:** SnO_2_, flexible, integrated anode, carbon nanofibers, Li-ion batteries

## Abstract

SnO_2_ is deemed a potential candidate for high energy density (1494 mAh g^−1^) anode materials for Li-ion batteries (LIBs). However, its severe volume variation and low intrinsic electrical conductivity result in poor long-term stability and reversibility, limiting the further development of such materials. Therefore, we propose a novel strategy, that is, to prepare SnO_2_ hollow nanospheres (SnO_2_-HNPs) by a template method, and then introduce these SnO_2_-HNPs into one-dimensional (1D) carbon nanofibers (CNFs) uniformly via electrospinning technology. Such a sugar gourd-like construction effectively addresses the limitations of traditional SnO_2_ during the charging and discharging processes of LIBs. As a result, the optimized product (denoted SnO_2_-HNP/CNF), a binder-free integrated electrode for half and full LIBs, displays superior electrochemical performance as an anode material, including high reversible capacity (~735.1 mAh g^−1^ for half LIBs and ~455.3 mAh g^−1^ at 0.1 A g^−1^ for full LIBs) and favorable long-term cycling stability. This work confirms that sugar gourd-like SnO_2_-HNP/CNF flexible integrated electrodes prepared with this novel strategy can effectively improve battery performance, providing infinite possibilities for the design and development of flexible wearable battery equipment.

## 1. Introduction

By virtue of their high energy density, long life span, and low environmental impact, Li-ion batteries (LIBs) are considered the predominant power source for many electronic applications, including portable devices, electric vehicles, and even future flexible wearable devices [1,2,3,4,5]. To meet the ever-growing market demands of high-energy LIBs, it is necessary to develop advanced anode materials to replace conventional graphite anode materials, which have a low specific capacity of 372 mAh g^−1^ and restricted energy density [6]. Therefore, there is a pressing need to develop a novel anode material to replace the traditional graphite.

In recent years, much attention has been paid to the search for alternative anodes with high reversible capacity. Among various MO_x_-based anode materials, tin dioxide (SnO_2_) stands out and has been followed with interest in the field of LIBs due to its high specific capacity (1494 mAh g^−1^), proper operating voltage, abundant resources, and low toxicity [7,8,9]. However, due to the severe volume expansion (over 300%) during the reciprocating motion of Li^+^, the electrode is severely crushed and the capacity is severely reduced, making the practical utility of SnO_2_ a major obstacle [10]. The low intrinsic electrical conductivity of this material will also result in a relatively inferior rate capacity in charging–discharging cycles [11,12]. As a result, the development of high-performance SnO_2_ anode materials has become essential for exploring next-generation LIBs.

Currently, many effective approaches have been proposed to address the aforementioned issues. For instance, it has been proposed SnO_2_ materials be synthesized at the nanoscale to effectively minimize volume-change stress and shorten the migration distance of Li^+^. Thus, considerable effort has been invested in the synthesis of nanoscale SnO_2_ with various morphologies, such as nanoparticles [13,14], nanotubes [15,16], nanorods [17,18], porous structures [19,20], and hollow structures [21,22,23]. For these special structures, an efficient approach is to construct electrode materials with hollow nanostructures, which provide sufficient space to cushion the large volume variation and inhibit the excessive growth of an unstable solid electrolyte interphase (SEI) [24]. Such cavities can provide additional space for the storage of Li^+^, thereby improving the specific capacity of Li-ion batteries. However, aside from the merits of hollow nanosized SnO_2_ structures, Li^+^’s low electronic conductivity contributes to poor cycling performance and inferior rate capability of the anode, which results in performance far below application standards. To address this obstacle, many researchers are committed to enhancing electrochemical performance via combining SnO_2_ with conductive substrates, such as carbon nanotubes [25,26], carbon nanosheets [27,28,29], foam copper [30,31], etc. Among these, one-dimensional (1D) carbon nanofibers (CNFs) have attracted widespread attention, owing to their advantages of high conductivity, large specific surface area, high flexibility and toughness, and good physical and chemical stability, making them an ideal substrate for compositing [32,33,34,35]. Such composite structures with high porosity not only further adapt to large volume changes but also facilitate effective charge transfer, thereby enhancing the overall conductivity of the electrode [36]. However, due to the weak binding (usually physical binding) between SnO_2_ and CNFs, the composite anode material is prone to poor cycling performance, which has significant limitations in alleviating significant volume changes.

Herein, we report a strategy combining a template method and electrospinning technology that can encapsulate SnO_2_ hollow nanospheres (SnO_2_-HNPs) in 1D CNFs (SnO_2_-HNP/CNFs) and directly use the synthesized flexible membranes as anodes for half/full Li-ion storage. This strategy perfectly combines SnO_2_-HNPs and 1D structure, presenting a sugar gourd-like shape as a whole, which realizes fast Li^+^/e^−^ transportation well with soft strain relaxation and strong conductivity. Without any auxiliary additives or current collectors, the obtained membrane can be directly and conveniently implemented as the working electrode, which not only significantly enhances energy density but also partially simplifies the experimental process. As a flexible and integrated anode material, SnO_2_-HNP/CNFs exhibit many significant electrochemical properties, including high reversible capacity, long-term cycling stability, and superior rate performance.

## 2. Experimental Section

### 2.1. Synthesis of SiO_2_

In a typical experiment, 60 mL of absolute ethanol, 3 mL of ammonia water, and 1 mL of deionized water were placed in a 100 mL flask, and after thorough mixing, 2.3 mL of tetraethyl orthosilicate was added dropwise. Then, this solution was stirred at a constant temperature of 20 °C for 6 h. After that, the resulting white precipitate was centrifuged and washed thoroughly three times with methanol, ethanol, and deionized water. Eventually, the obtained product was dried under vacuum (60 °C, 12 h).

### 2.2. Synthesis of SiO_2_@SnO_2_

For the synthesis of SiO_2_@SnO_2_, 0.05 g of SiO_2_ was dispersed in 3 mL of ethanol and 3 mL of deionized water to form a mixed solution. Then, 0.15 g of K_2_SnO_3_·3H_2_O and 0.045 g of urea were dispersed in the above solution. After ultrasonic stirring for 30 min, the stirred milky solution was transferred to a 50 mL of polytetrafluoroethylene-lined stainless-steel autoclave and kept at 150 °C for 24 h. After the reaction, the milky white product was obtained by centrifugation, washed at least three times with deionized water and ethanol, and then vacuum-dried at 60 °C for 12 h.

### 2.3. Synthesis of SnO_2_-HNPs

In order to obtain the hollow structure, we needed to process the obtained SiO_2_@SnO_2_ product through etching. The specific operation process is as follows: 200 mg of SiO_2_@SnO_2_ was dispersed in 2 mol/L NaOH solution in an oil bath to remove the inner SiO_2_ spheres (50 °C, 8 h). After cooling down to room temperature, it was washed thoroughly with deionized water until neutral, and dried under vacuum (60 °C, 12 h).

### 2.4. Synthesis of SnO_2_-HNP/CNFs

The SnO_2_-HNP/CNFs were designed through the electrospinning method. Briefly, SnO_2_-HNPs with different content (100, 200, and 300 mg) were stirred to dissolve in 5 mL of N, N-Dimethylformamide, during which process 0.4 g of polyacrylonitrile was added. After stirring for 12 h, the resulting viscous solution was loaded and the electrospun nanofibers (SnO_2_-HNP/NFs) were collected through aluminum foil (15 kV, 18 cm). Subsequently, the obtained SnO_2_-HNP/NFs were firstly stabilized in a tube furnace under air atmosphere (250 °C, 1 °C min^−1^, 3 h), in the train of the obtained brown nanofibers were heated at 600 °C for 3 h (2 °C min^−1^) under N_2_ atmosphere. The calcined products were denoted as SnO_2_-HNP/CNF-1, SnO_2_-HNP/CNF, and SnO_2_-HNP/CNF-3 according to the different content of SnO_2_-HNPs. Moreover, during the process of assembling Li-ion batteries, we put the flexible film (SnO_2_-HNP/CNF) into a mortar and ground it until crushed, naming it as SnO_2_-HNP/CNF-G.

### 2.5. Material Structure Characterization

The crystal structure of SnO_2_-HNP/CNFs was measured by X-ray powder diffractometer (XRD) patterns recorded on a diffractometer with Cu Ka radiation (Rigaku, Dmax2500, Tokyo, Japan). Raman spectra were recorded on a model confocal microscopy Raman spectrometer (Renishaw, RM2000, Gloucestershire, UK). X-ray photoelectron spectroscopy (XPS) was measured by X-ray instrument (Thermo Fischer, ESCALAB 250, Waltham, MA, USA). The morphology of the SnO_2_-HNP/CNFs was displayed by field emission scanning electron microscopy (SEM, Hitachi, S-4800, Chiyoda-ku, Japan). The specific surface area was calculated by analyzing the nitrogen adsorption–desorption isotherms of Brunauer–Emmett–Teller (BET) at 77 k (Micromeritics instrument Ltd., Tristar 3000, Norcross, GA, USA).

### 2.6. Electrochemical Measurement

In electrochemical experiments, we directly assembled the prepared SnO_2_-HNP/CNFs film as the free-standing anode electrode materials for Li-ion batteries, and the mass of the flexible electrode was about 1.5 mg. In addition, as the entire process was carried out under laboratory conditions, we chose coin cells (CR2025) for actual battery testing, including charge–discharge performance testing, rate performance testing, cycle stability testing, etc. This is because coin cells have the advantages of small size, high voltage, and relatively stable performance. The coin cells are composed of the battery shell, the prepared SnO_2_-HNP/CNFs anode electrode, the electrolyte (1 M LiPF_6_ in ethylene carbonate/dimethyl carbonate (EC/DMC, 1:1 vol%), the separator (Celgard 2400 membrane), the gasket, the spring piece, and the lithium metal as the counter electrode. Finally, we assembled all parts of the coin cells through the vacuum glovebox, and tested their battery performance using the electrochemical workstation. The specific testing parameters are as follows: the galvanostatic charge/discharge experiments were performed with a cutoff voltage window of 0.01–3.0 V using a Land Battery Measurement System (Land, Wuhan, China). The cyclic voltammetry (CV) was performed using a CS350 Electrochemical Workstation (Wuhan CorrTest Instruments Co., Ltd., Wuhan, China)

## 3. Results and Discussion

**Figure 1:**
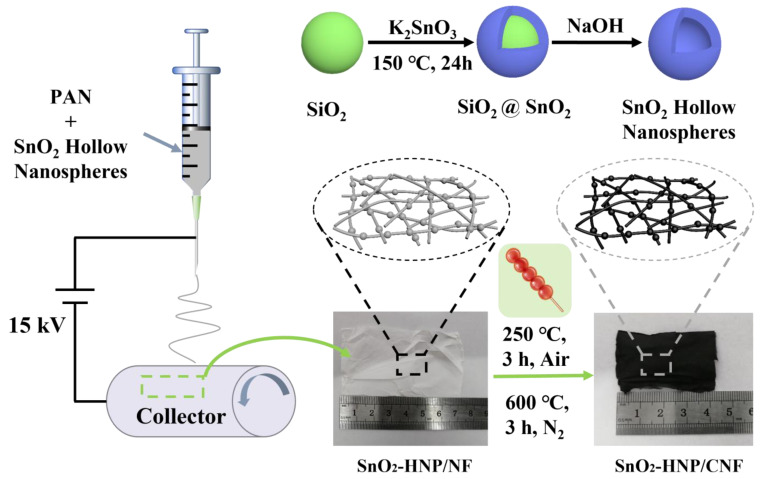
Synthesis diagram of SnO_2_-HNP/CNFs.

The overall synthesis process of SnO_2_-HNP/CNFs is depicted in Figure 1. In the beginning, we prepared monodisperse SiO_2_ nanospheres by the sol–gel method, and then employed them as the hard template to prepare SnO_2_-HNPs via a hydrothermal reaction. At this moment, the sugar gourd-like-shaped “gourd” part has been prepared. Subsequently, we take monodisperse SnO_2_-HNPs as the precursor, spin them evenly into nanofibers (SnO_2_-HNP/NF) via the electrospinning technology, and then prepare SnO_2_-HNP/CNF through subsequent heat treatment technology. In this way, SnO_2_-HNPs and CNFs are tightly combined, so that a complete structure of sugar gourd-like shape has been successfully prepared.

**Figure 2:**
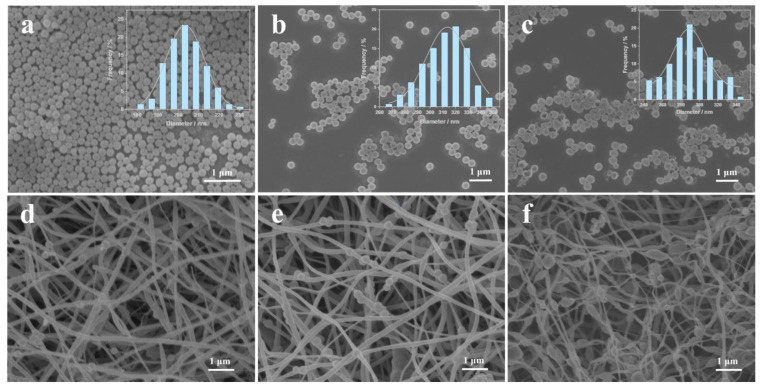
SEM images of (**a**) SiO_2_, (**b**) SiO_2_@SnO_2_, (**c**) SnO_2_-HNPs, (**d**) SnO_2_-HNP/CNF-1, (**e**) SnO_2_-HNP/CNF, and (**f**) SnO_2_-HNP/CNF-3.

The microstructures of various products are observed by SEM. Figure 2a shows that the morphology of SiO_2_ nanospheres is relatively uniform, with particle sizes ranging from 180 to 230 nm, with 205 nm SiO_2_ nanospheres accounting for the majority. Figure 2b clearly displays that the morphology of SiO_2_@SnO_2_ nanospheres has been maintained, but the difference is that their particle size has increased (ranging from 265 to 350 nm, with 320 nm accounting for the majority). Figure 2c further illustrates that the spherical shape and the particle size of SiO_2_@SnO_2_ nanospheres after etching remain basically unchanged, indicating that the SiO_2_ inside has been etched off and the SnO_2_ hollow spherical structure has been successfully synthesized. Moreover, the products with different SnO_2_-HNPs contents are shown in Figure 2d,e. It can be seen that in SnO_2_-HNP/CNF, only a small amount of SnO_2_-HNPs is encapsulated in the CNFs, and the CNFs occupy the majority of the material (Figure 2d). Differently, it can be clearly seen that the SnO_2_-HNPs have been evenly distributed in 1D CNFs (SnO_2_-HNP/CNF), showing a sugar gourd-like shape (Figure 2e). The SnO_2_-HNP/CNF-3 product shown in Figure 2f exhibits an uneven morphology, with an excess of SnO_2_-HNPs loaded on each carbon nanotube. It is obvious that a large number of SnO_2_-HNPs are concentrated in one position, resulting in each carbon nanotube becoming finer and more prone to fracture. Moreover, energy-dispersive spectrometry (EDS) of SnO_2_-HNP/CNF was conducted to confirm the elemental composition, demonstrating the existence of C, N, O, and Sn elements (Figure S1). The results preliminarily confirm the formation of Sn-based oxides into the nitrogen-doped carbon nanofibers.

**Figure 3:**
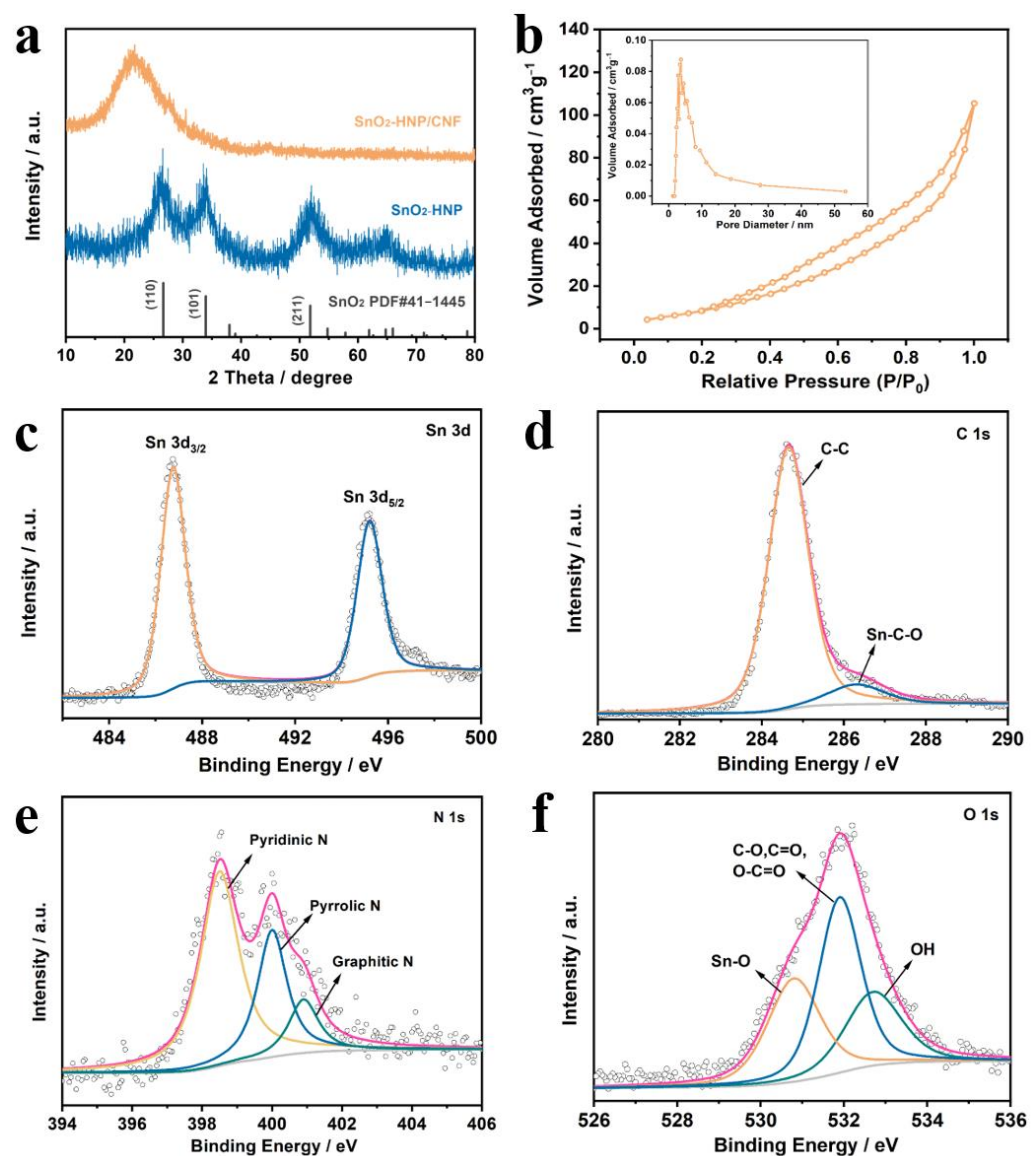
(**a**) XRD patterns of SnO_2_-HNP and SnO_2_-HNP/CNF. (**b**) N_2_ adsorption−desorption isotherms and the pore size distribution (inset) of SnO_2_-HNP/CNF. High-resolution XPS spectra of Sn 3d (**c**), C 1s (**d**), N 1s (**e**), and O 1s (**f**) for SnO_2_-HNP/CNF.

Figure 3a presents the XRD patterns of SnO_2_-HNP and SnO_2_-HNP/CNF. Obviously, SnO_2_-HNPs show a set of characteristic peaks at 26.6°, 33.8°, and 51.8°, corresponding to (110), (101), and (211) planes of the SnO_2_ (PDF#41-1445), respectively. However, the SnO_2_-HNP/CNF only displays one peak at ~24° attributed to the amorphous carbon. This situation is due to the carbon peak of the outermost CNFs masking the peak of SnO_2_-HNP within its structure, implying the formation of tightly packed structures. Moreover, the carbon structure property of SnO_2_-HNP/CNF is determined by Raman spectroscopy (Figure S2). Based on the relative intensity ratio (I_D_/I_G_ = 1.05) of the D and G peaks, it is determined that SnO_2_-HNP/CNF contains abundant structural defects [37,38]. Such defect structure may be caused by the doping of N elements in composite material. Additionally, Brunauer–Emmett–Teller (BET) testing was also conducted to further investigate the porous structure of SnO_2_-HNP/CNF (Figure 3b). The calculated BET value is 85 m^2^ g^−1^, while the average pore range is approximately distributed within the range from 2 to 12 nm and concentrated at 3.80 nm. The larger BET area with abundant pores of SnO_2_-HNP/CNF film is conducive to the transfer of Li^+^ during battery charging/discharging as the integrated anode for Li-ion batteries [39]. To evaluate the mechanical strength, the stress–strain curve of SnO_2_-HNP/CNF flexible films is conducted (Figure S3). The SnO_2_-HNP/NF displays the maximal tensile break strength of 0.93 MPa and a tensile strain of 6.14%, while the SnO_2_-HNP/CNF exhibits the maximal tensile break strength of 0.44 MPa and a tensile strain of 3.27%. This indicates that the calcination process enabled the SnO_2_-HNP/CNF film to not only obtain excellent electrical conductivity but still maintain good mechanical strength, making it possible to serve as a flexible integrated anode.

To identify the chemical compositions and surface electronic structure of the SnO_2_-HNP/CNF, X-ray photoelectron spectroscopy (XPS) analysis was performed. Full-range XPS survey spectrum directly indicates the existence of Sn, C, N, and O in the SnO_2_-HNP/CNF (Figure S4), which is consistent with the EDS data. The presence of the peak without Si indicates that the SiO_2_ as a template has been completely etched. As shown in Figure 3c, the Sn 3d segment of XPS comes from the Sn-O bond in SnO_2_ lattice. The peaks occurred at 486.7 and 495.2 eV are corresponded to Sn 3d_3/2_ and Sn 3d_5/2_ spins, separately, with an energy separation of 8.5 eV confirming the valence state of the Sn atom in SnO_2_ (Sn^4+^ oxidation state) [40,41]. This also indirectly confirms the existence of SnO_2_ in the composites. The C 1s spectrum (Figure 3d) is divided into two peaks at 284.6 and 286.3 eV, corresponding to C-C bonds and Sn-C-O bonds, respectively. The bonding structure of nitrogen has a great influence on the properties of nitrogen-doped carbon materials, the types of nitrogen atoms doped in SnO_2_-HNP/CNF are examined. According to the spectra in Figure 3e, three different nitrogen species of N 1s can be observed, and the peaks at 398.5, 400.0, and 400.9 eV are consistent with pyridinic N, pyrrolic N, and graphitic N, respectively [42]. Among them, graphite N is located in the network structure of carbon, which is formed by nitrogen atoms replacing the carbon atoms in the graphite layer. It has the same configuration as graphite carbon, which can improve the conductivity of the carbon material. The pyridine N in the material can provide more active sites, which are beneficial for the storage of Li^+^ ion. Thereby the conductivity of the SnO_2_-HNP/CNF can be further improved [43,44]. As to the O 1s spectrum (Figure 3f), the three peaks centered at binding energies of 530.8, 531.9, and 532.7 eV are assigned to the Sn-O, C-O/C=O/O-C=O, and OH bonds, respectively [45]. Furthermore, to measure the content of SnO_2_ in SnO_2_-HNP/CNF, we conducted thermogravimetric testing (TG), and the results are shown in Figure S5. There are two weight-loss processes in SnO_2_-HNP/CNF during the heating process. The first weightlessness process occurs below 300 °C (4%), corresponding to the dehydration process of the material. The second weight-loss process occurs between 300 and 480 °C (47%), as the CNFs in the composite are oxidized into CO_2_. Finally, the TG curve remains at around 49%, indicating that the SnO_2_ content in the material is around 49%.

**Figure 4:**
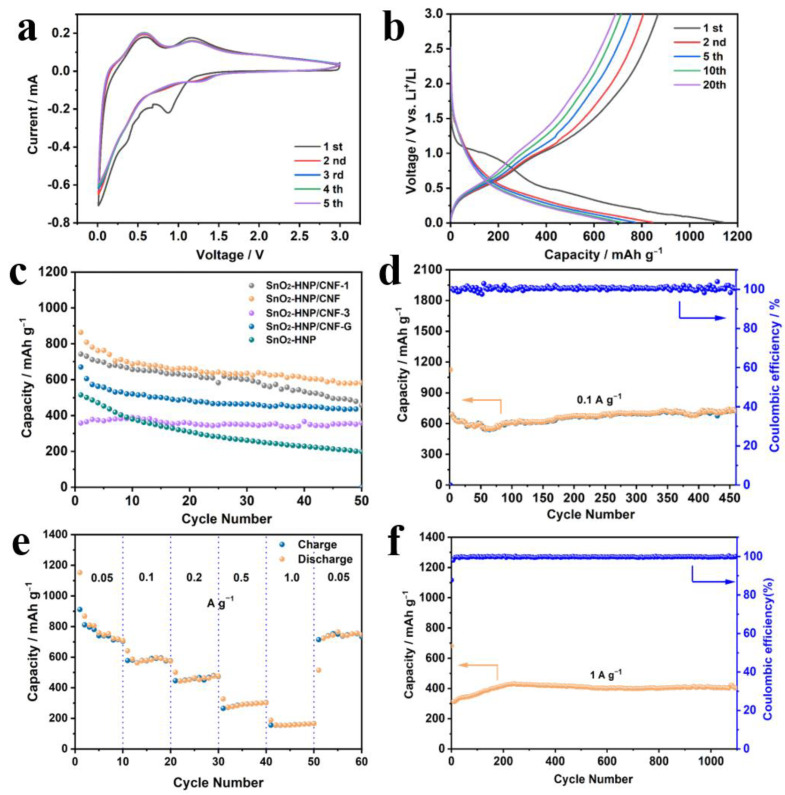
Electrochemical performance of SnO_2_-HNP/CNF in half LIBs. (**a**) CV curves at 0.1 mV s^−1^. (**b**) Charge/discharge curves at 0.05 A g^−1^. (**c**) Cycling performance of various products at 0.05 A g^−1^. (**d**) Cycling performance of SnO_2_-HNP/CNF at 0.1 A g^−1^. (**e**) Rate performance at different current densities. (**f**) Cycling performance of SnO_2_-HNP/CNF at 1 A g^−1^.

To evaluate the electrochemical behaviors of SnO_2_-HNP/CNF as anode materials for LIBs, the cyclic voltammetry (CV) curves were constructed using coin cells between 0.01 and 3 V (Figure 4a). The irreversible peak at 0.87 V appears in the first cycle and disappears during subsequent cycles. This is because the solid–electrolyte interphase (SEI) layer is formed during the first cycle. With further discharge, the reduction peak occurs at 0.35 V as a result of lithium alloying with Sn and forming Li_x_Sn. The oxidation peaks at 0.57 and 1.15 V are attributed to the dealloying of Li_x_Sn and the transition of Sn to SnO_2_, respectively. In addition, the following scan displays similar and overlapping CV curves, indicating the excellent reversibility of the alloying reaction between Sn and Li. Figure 4b exhibits the representative charge/discharge curves of SnO_2_-HNP/CNF (0.05 A g^−1^). In the initial discharge cycle, two voltage platforms in the charge/discharge curve correspond to the two reduction voltages in the CV curve, respectively. Moreover, the first discharge and charge capacities of SnO_2_-HNP/CNF reach 1136.8 and 867.1 mA h g^−1^, respectively. The formation of the SEI and the decomposition of the electrolyte are main causes of the capacity decay during the initial cycle. For comparison, the cycling performance of different content of SnO_2_ is investigated in Figure 4c. The SnO_2_-HNP/CNF displays the highest specific capacity (~584.3 mAh g^−1^ at 0.05 A g^−1^) and the most stable cycling performance (50 cycles), higher than those of SnO_2_-HNP/CNF-1 (457.2 mA h g^−1^) and SnO_2_-HNP/CNF-3 (352.1 mA h g^−1^). Moreover, the capacity differences between the SnO_2_-HNP (200.9 mA h g^−1^) and SnO_2_-HNP/CNF also demonstrate the superior performance of the composite film. Also, the SnO_2_-HNP/CNF-G shows a relatively low capacity (413.4 mA h g^−1^), which is associated with the addition of the PVDF binder. Different from an integrated electrode, grinding with the PVDF binder can induce the expansion of electrodes, causing irreversible capacity losses. Figure 4d illustrates the cycling performance of SnO_2_-HNP/CNF at 0.1 A g^−1^ current density. The reversible capacity is 692.4 mA h g^−1^ after 450 cycles, with a capacity retention rate of 94.2% relative to the second cycle (735.1 mA h g^−1^). Noteworthy, the capacity has a slight decay in the first 50 cycles and then picks up, which corresponds to the typical characteristics of metal oxide in the storage lithium processes. The reasons can be traced back to the reversible formation of a polymeric gel-like layer through electrolyte decomposition and the improvement of Li^+^ accessibility during further cycling [46]. The rate properties are also evaluated and shown in Figure 4e. The reversible capacities of SnO_2_-HNP/CNF are759.5, 590.8, 467.9, 300.1, and 164.1 mAh g^−1^ at 0.05, 0.1, 0.2, 0.5, and 1.0 A g^−1^, respectively. It is noteworthy that when the current density was reset to 0.05 A g^−1^, the capacity could recover to a high specific capacity of 753.1 mAh g^−1^, comparable to the initial discharge capacity, indicating that SnO_2_-HNP/CNF has good rate performance as the integrated flexible anode. The long-term cycling of SnO_2_-HNP/CNF exhibits that the capacity is always stable at 410.2 mAh g^−1^ without any degradation after continuous operation over 1000 cycles at the large current density of 1 A g^−1^ (Figure 4f).

**Figure 5:**
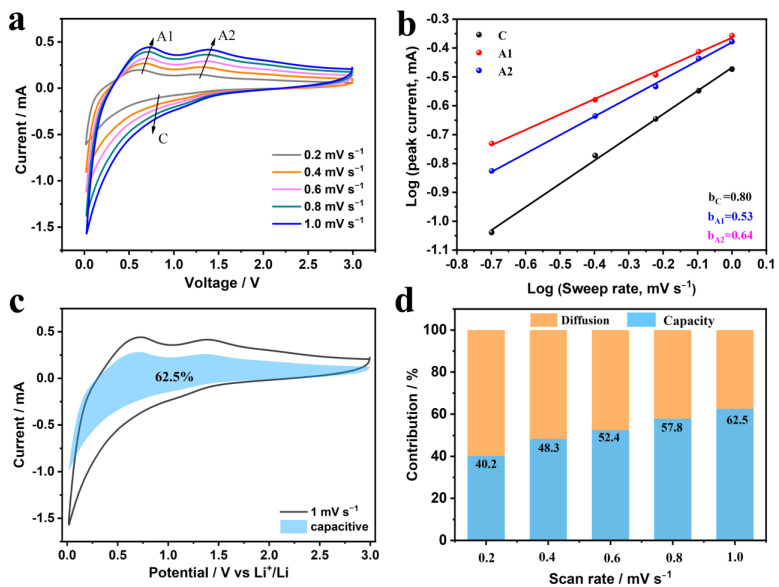
Kinetic analysis of SnO_2_-HNP/CNF in half LIBs. (**a**) CV curves at different scan rates. (**b**) b−value using the relationship between peak current and scan rate. (**c**) Capacitive contribution at 1 mV s^−1^. (**d**) Contribution ratio of capacitive at various scan rates.

The electrochemical reaction kinetics of SnO_2_-HNP/CNF were also investigated in detail to reveal the underlying mechanisms for outstanding performance. The CV curves at scan rates of 0.2–1 mV s^−1^ and the slope b values of each redox peak calculated by the equation: *log i = blog ν + log a* are displayed in Figure 5a,b [47,48,49]. The b value between 0.5 and 1 implies that the electrochemical capacity of SnO_2_-HNP/CNF is contributed by both pseudocapacitive and diffusion behaviors. Besides, the capacitive contribution at the scan rate of 1 mV s^−1^ is calculated and fitted by the equation: *i = k*_1_*ν + k*_2_*ν*^1/2^ [50,51]. It can be seen that the capacitive contribution at 1 mV s^−1^ is determined to be 62.5% (Figure 5c). Noteworthy, the capacitance contribution (from 40.2% to 62.5%) increases along with the scan rate increases (from 0.2 to 1 mV s^−1^) (Figure 5d). This reveals that at low current densities, the electrochemical behavior primarily exhibits diffusion control process, while it transforms into a capacitance-dominated control process in rapid charging and discharging, which contributes to excellent high-rate lithium storage performance.

**Figure 6:**
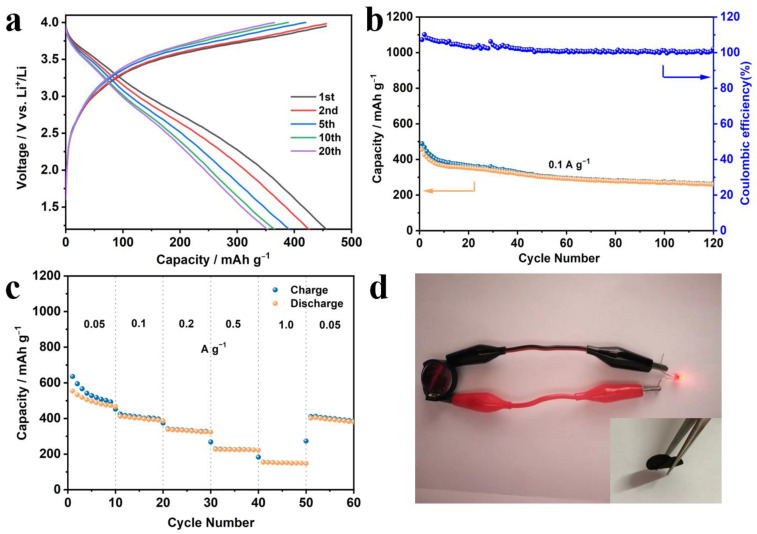
Electrochemical performance of SnO_2_-HNP/CNF in full LIBs. (**a**) Charge/discharge curves and (**b**) cycling performance at 0.1 A g^−1^. (**c**) Rate performance at different current densities. (**d**) Coin full battery lighting electronic thermometer.

To verify the practical value of SnO_2_-HNP/CNF, we also assembled and evaluated lithium-ion full-cell with SnO_2_-HNP/CNF as the anode and LiCoO_2_ as the cathode. The initial discharge and charge capacities of the full cell exhibit 455.8 and 455.3 mAh g^−1^, respectively, and there was no significant decay in the subsequent cycles (Figure 6a). It also shows a favorable cycling stability at 0.1 A g^−1^, with a reversible capacity of 265.7 mAh g^−1^ after 120 cycles, corresponding to a capacity retention rate of 65.4% and a coulombic efficiency of 100% (Figure 6b). What is more, the rate performance of the full cell based on SnO_2_-HNP/CNF was also examined (Figure 6c). At a high current density of 1 A g^−1^, the full cell based on SnO_2_-HNP/CNF still has a reversible capacity of 153.4 mAh g^−1^. Similar to the half-cells, the capacity can be restored to the initial state when the current density decreases, illustrating the excellent rate performance of SnO_2_-HNP/CNF. The excellent electrochemical lithium storage performance of half- and full-cells with SnO_2_-HNP/CNF as the flexible integrated anode can be attributed to the synergistic effect of carbon nanofibers and SnO_2_-HNP. Where SnO_2_ provides high capacities, the unique hollow structure and the encapsulation of carbon nanofibers weaken the capacity decay due to the volume expansion of SnO_2_, which contributes to the long-cycle stability. Not only that, the addition of flexible carbon nanofiber also avoids the use of binder and increases the electrical conductivity, ensuring the charging and discharging performance at the high current density. Owing to the above excellent performance, we conducted the LED light-up test. The flexible SnO_2_-HNP/CNF electrode is shown in the illustration in Figure 6d, demonstrating its good flexibility and mechanical properties. The Li^+^ coin full-cell assembled by SnO_2_-HNP/CNF flexible electrodes can also successfully light up LED lights, which verifies its practical application potential.

## 4. Conclusions

In summary, a novel SnO_2_-HNP/CNF flexible integrated electrode has been designed by combining the template method and electrospinning technology for half and full LIBs. The combination of 1D nanofibers and SnO_2_ hollow structure forms a sugar gourd-like shape, which not only alleviates the volume expansion and limits the growth of the SEI membrane but also enhances the overall cycling performance of electrode materials. Also, without any auxiliary additives and current collectors, the obtained membrane can be directly and conveniently implemented as the working electrode, which not only signally enhances the energy density but also partially simplifies the experimental process. Based on the above advantages, the optimized SnO_2_-HNP/CNF delivers excellent half/full LIBs performance. It is believed that this synthetic strategy and morphology design could be extended to the construction of other MO_x_-encapsulated carbon nanofibers with 3D hollow structures as the binder-free integrated anode for various energy-related applications.

## Data Availability

The data is available on reasonable request from the corresponding author.

## Supplementary Materials

The following supporting information can be downloaded at https://www.mdpi.com/article/10.3390/nano13152226/s1. Figure S1: Energy-dispersive spectrometry (EDS) of SnO_2_-HNP/CNF; Figure S2: Raman spectra of SnO_2_-HNP/CNF; Figure S3: Stress–strain curve of SnO_2_-HNP/CNF and SnO_2_-HNP/NF; Figure S4: XPS survey of SnO_2_-HNP/CNF; Figure S5: Thermogravimetric analysis (TGA) of SnO_2_-HNP/CNF under air with a ramp rate of 5 °C min^−1^.
