# Supplementary material for: Construction of Carbon Nanofiber-Wrapped SnO2 Hollow Nanospheres as Flexible Integrated Anode for Half/Full Li-Ion Batteries
Source: Nanomaterials (Basel). 2023 Jul 31;13(15):2226. doi: 10.3390/nano13152226 (PMC10421331; doi:10.3390/nano13152226)
Supplement: Supplementary file 1 [file nanomaterials-13-02226-s001.zip › nanomaterials-2513581-supplementary.pdf]

nanomaterials

Supporting Information

Construction of Carbon Nanofiber-Wrapped SnO2 Hollow Nanospheres as Flexible Integrated Anode for Half/Full Li-Ion Batteries

Qi Shao [1], Jiaqi Liu [2,3], Xiantao Yang [1], Rongqiang Guan [1], Jing Yu [1] and Yan Li [1,2,*]

1 School of Electrical and Information, Jilin Engineering Normal University, Changchun 130052, China; shaoqi727@163.com (Q.S.)
2 School of Materials Science and Engineering, Changchun University of Science and Technology, Changchun 130022, China; liujiaqi_forsci@163.com
3 School of Metallurgy, Northeastern University, Shenyang 110819, China
* Correspondence: liyan_forsci@163.com; Tel.: +86-431-8179-0066

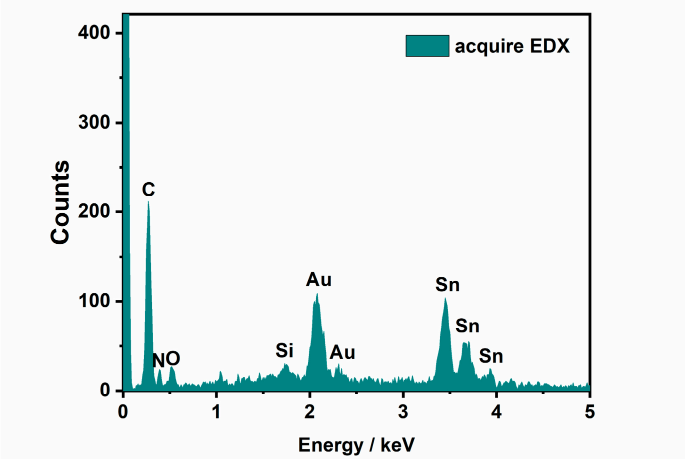

Figure S1. Energy–dispersive spectrometry (EDS) of SnO_2-HNP/CNF.

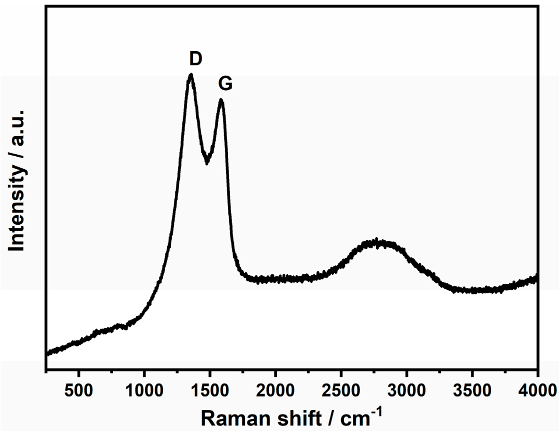

Figure S2. Raman spectra of SnO_2-HNP/CNF.

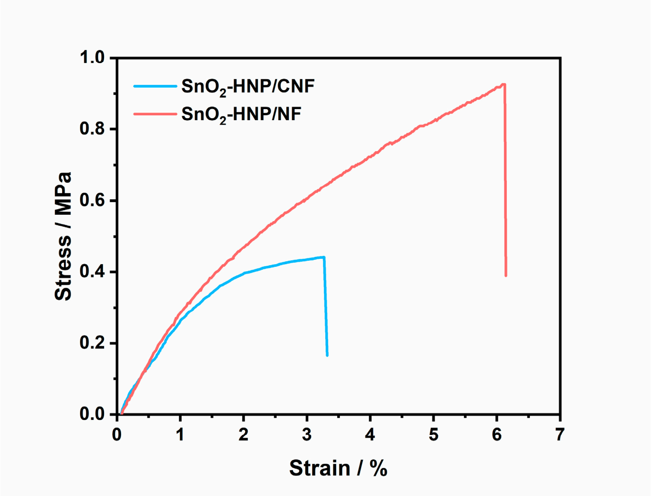

Figure S3. Stress−strain curve of SnO_2-HNP/CNF and SnO_2-HNP/NF.

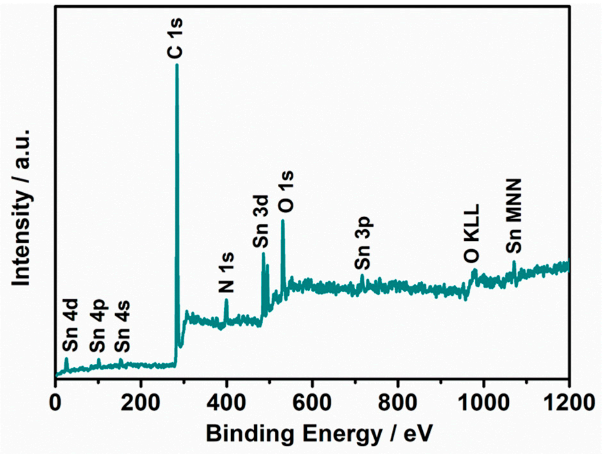

Figure S4. XPS survey of SnO_2-HNP/CNF.

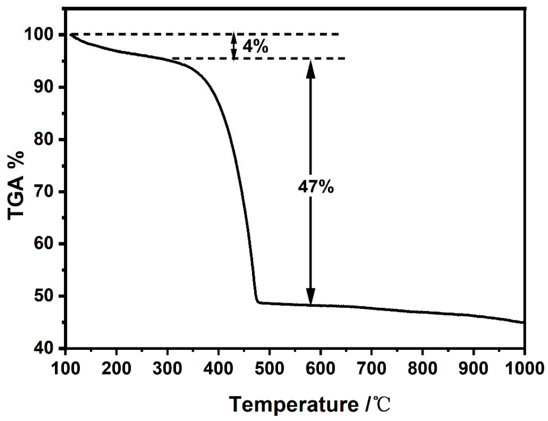

Figure S5. Thermogravimetric analysis (TGA) of SnO_2-HNP/CNF under air with a ramp rate of 5 °C min^{-1}.
